# Clinical characteristics of nephrocalcinosis in a tertiary children's hospital

**DOI:** 10.3389/fped.2025.1672632

**Published:** 2025-11-12

**Authors:** Jiajia Zheng, Jie Cao, Lan Chen, Xuhua Xia

**Affiliations:** Department of Medical General Ward, National Clinical Research Center for Child Health and Disorders, Ministry of Education Key Laboratory of Child Development and Disorders, Chongqing Key Laboratory of Pediatric Metabolism and Inflammatory Diseases, Children’s Hospital of Chongqing Medical University, Chongqing, China

**Keywords:** nephrocalcinosis, children, etiology, ultrasonography, genetic test

## Abstract

**Background:**

Nephrocalcinosis (NC) is often associated with prematurity, genetic, and/or metabolic disorders. However, studies focusing on NC in pediatric population remain limited.

**Aims:**

This study aimed to explore the etiology of NC and characterize clinical manifestation in children.

**Methods:**

We retrospectively reviewed the electronic medical records of consecutive 50 children diagnosed with NC between January 1, 2016, and December 31, 2022, at the Children's Hospital of Chongqing Medical University. The data were analyzed to determine the underlying causes and clinical presentations of NC.

**Results:**

Of the 50 children diagnosed with NC, 50% were younger than 2 years old. Congenital diseases were diagnosed in 54% of the children, with renal tubular acidosis (RTA) accounting for 44% of these cases. Genetic testing confirmed diagnoses in 45% of cases (10 out of 22 tested). Although NC often presents with non-specific and diverse symptoms, 18% of the children were asymptomatic. During a long-term follow-up, 69% of cases showed no signs of improvement and 2 patients later developed nephrolithiasis. Only 13% suffered from impaired renal function and one of them developed into chronic kidney disease.

**Conclusions:**

Congenital or genetic disorders are the primary causes of NC in children. Most cases persist overtime, underscoring the need for early identification of underlying causes through genetic testing and the development of more effective treatments for NC. While most children maintained normal renal function, the relationship between NC and renal functional decline warrants further investigation.

## Introduction

Nephrocalcinosis (NC) involves the deposition of calcium in the renal tubules and interstitium, resulting from a variety of hereditary and acquired diseases (1, 2). In contrast, nephrolithiasis (NL) describes solid stones within the renal collection system (3). Both NC and NL are increasingly recognized as risk factors of chronic kidney disease (CKD) and kidney failure (4). NC manifests in distinct ultrasonographic patterns depending on the anatomic area involved, categorized as either cortical and diffuse NC or medullary NC. The latter is further divided based on echogenicity into grades I–III (5).

Despite increasing recognition, precise prevalence and incidence rates of NC remain unknown. However, evidence suggests that the annual incidence of pediatric NL has been rising by about 6%–10% in recent decades (6, 7). A notable increase in pediatric urolithiasis hospitalization rates has been documented across 41 pediatric hospitals, ranging from 1.5- to 11-fold (8).

Unlike adults, in whom environmental factors are the predominant contributors to kidney stones, the primary drivers of NC in children are preterm birth, genetic, and metabolic disorders (9, 10). Gefen et al. reported that the most common diagnoses were hereditary hypophosphatemic rickets with hypercalciuria (HHRH), followed by primary hyperoxaluria type I (PH type I) (11). Similarly, Daga et al. identify primary hyperoxaluria type I, renal tubular acidosis (RTA), Bartter syndrome, and infantile hypercalcemia as key conditions linked to childhood NC by whole exome sequencing (12). The prognosis of NC is primarily influenced by its underlying causes (2) and it is closely linked to serious morbidities, including CKD, metabolic bone disease (MBD), and end-stage renal disease (ESRD) (13, 14).

Despite increasing recognition of NC, comprehensive studies on its prevalence, etiology, and long-term outcomes in pediatric patients are sparse. To address this gap, our study undertook a retrospective analysis of children diagnosed with NC at the Children's Hospital of Chongqing Medical University over a seven-year period. The primary aims of this research were to elucidate the etiology and clinical characteristics of NC in the pediatric population, as well as to evaluate the interventions and prognoses associated with this condition.

## Methods

This study retrospectively collected and analyzed the medical records of children diagnosed with NC who were admitted to the Children's Hospital of Chongqing Medical University between January 1, 2016, and December 31, 2022. We enrolled patients with sonographic reports of “nephrocalcinosis”or “medullary nephrocalcinosis” by radiologists and excluded patients with solitary nephrolithiasis. Patients were excluded from the study if their key general demographic records were incomplete. A total of 50 children met the inclusion criteria and were included in the analysis.

Comprehensive data were meticulously recorded, including demographic details (such as sex and age), clinical manifestations, laboratory and imaging results, interventions undertaken, and patient prognoses. The study protocol was approved by the Institutional Review Board of the Children's Hospital, Chongqing Medical University, ensuring adherence to ethical standards in research.

## Definitions

NC was diagnosed by ultrasonography or computer tomography (CT) (10). In the ultrasound description, linear calcifications of the renal cortex in the form of a band that extended to the septal cortex was diagnosed as cortical NC, and multifocal scattered echogenic foci at the medulla or a diffuse hyperechoic medulla was diagnosed as medullary nephrocalcinosis (1, 15). In the axial non-contrast CT section of the kidneys, multiple punctate high-density, linear or “band” hyperdensity were diagnosed as cortical NC, and a linear echogenic focus at the tip of the renal pyramid along the margins of the fornix were diagnosed as medullar NC (1, 15). The disappearance of NC as described by ultrasonography or CT was defined as NC improvement. CKD was classified according to the clinical practice guideline for the evaluation and management of CKD (16). Methods for renal volume and length measurement were previously described (17)*.* Bone changes included bone fractures, reduced bone density, and osteoporosis.

## Statistical analysis

Continuous variables were expressed as mean ± standard deviation (SD) for normally distributed data, or as median and interquartile range (IQR) for non-normally distributed data. Categorical variables were presented as counts and percentages. In the sub-group analysis, comparisons between the three groups were performed using the Kruskal–Wallis test for continuous variables and Fisher's exact test for categorical variables. *P* < 0.05 indicated statistical significance. All collected data were analyzed using SPSS software, version 19.0.

## Results

### Demographic and clinical characteristics

A total of 50 children diagnosed with NC were included. As shown in Table 1, most of the children (50%) were younger than 2 years old, with 36% being between 1 and 12 months of age. Preterm birth was noted in 10% of the patients. Among them, only one patient was diagnosed with NC in infancy, and the other 4 patients were diagnosed over 4 years of age. Only 28% of the children were diagnosed with NC due to kidney-related manifestations (including seeking for medical advice due to kidney-related symptoms and abnormal findings on renal ultrasound), and the majority of them were incidentally diagnosed.

**Table 1:** Demographic features and clinical parameters for children admitted for NC.

Variable	Category	*N* (%)
Preterm infant		5 (10)
Age	<1 month	1 (2)
	1–12 months	18 (36)
	1–2 years	6 (12)
	3–5 years	10 (20)
	6–12 years	15 (30)
Sex	Male	31 (62)
	Female	19 (38)
Family history of renal disease		10 (20)
Clinical presentation	Failure to thrive	25 (50)
	Dehydration	4 (8)
	Nausea/vomiting	14 (28)
	Abdominal distension	4 (8)
	Constipation	2 (4)
	Abdominal pain	6 (12)
	Polyuria	5 (10)
	Acute renal insufficiency	18 (36)
	Hematuria	12 (24)
	Proteinuria	13 (27)
Urine calcium/creatinine >0.21 mmol/L/mmol/L		16 (80)
Low 24 h urinary calcium (mmol)		6 (30)
Low 24 h urinary phosphorus (mmol)		14 (88)
Urinary tract infection		19 (39)
Hemoglobin <90 g/L		9 (18)
pH <7.35 mmol/L		18 (44)
Corrected total serum calcium (mmol/L)	<2.2	12 (24)
	2.2–2.6	26 (52)
	>2.6	12 (24)
PTH (pg/mL)	≤69	24 (89)
	>69	3 (11)
Low levels of 25(OH)D3 (nmol/L)		10 (38)
Bone changes		8 (30)
Cardiovascular system	Sinus tachycardia	9 (45)
	T-wave alternans	8 (40)
	ST-T changes	4 (20)
Diagnosed by imaging	Ultrasonography	48 (96)
	CT	2 (4)
NC type	Medullary	42 (84)
	Cortical	8 (16)
Bilateral renal involvement		46 (92)
Abnormal kidney volume		5 (10)
Kidney structural abnormalities		24 (48)

NC, nephrocalcinosis; PTH, parathyroid hormone.

Ten cases (20%) had a family history of renal disease, including NL (70%), NC (10%), renal cysts (10%) and hypercalciuria (10%). Among these cases, the mother of one child concurrently suffered from RTA, polycystic kidney disease, and NL. Only 3 out of 10 children with positive family history underwent genetic analysis: 2 had SLC4A1 gene mutation and 1 had PKHD gene mutation.

Clinically, failure to thrive was the most common presentation, observed in 50% of the cases, followed by acute renal insufficiency (36%), and nausea-vomiting (28%). Hematuria and proteinuria were present in 24% and 27% of the patients, respectively. Notably, 80% of the patients had elevated urine calcium/creatinine ratios, while 88% had low 24 h urinary phosphorus levels. Findings of electrocardiogram such as sinus tachycardia and T-wave alternans, were observed in 45% and 40% of the patients, respectively.

Ultrasonography was the primary diagnostic imaging modality, used in 96% of the cases, and revealed medullary NC in 84% of the patients. Bilateral renal involvement was documented in 92% of the cases, and 48% of the patients had kidney abnormalities.

Blood calcium is closely related with NC. Sub-group analysis was done regarding total serum calcium level as summarized in Table 2. After treatment, the corrected serum calcium (CSC) of all patients returned to normal. However, during follow-up, the improvement rate of NC in the normal CSC group, hypercalcemia group, and hypocalcemia group were 72.7%, 80%, and 60%, respectively. Two cases in the hypocalcemia group developed NL during the follow-up.

**Table 2:** Sub-group comparison analysis based on corrected total serum calcium.

Variable	Normal CSC (*N* = 26)	Hypercalcemia (*N* = 12)	Hypocalcemia (*N* = 12)	*P* value
Corrected total serum calcium, mmol/L	2.4 [2.3–2.5]	4.1 [3.6–4.4]	2.1 [2.0–2.1]	<0.001
Serum sodium, mmol/L	137.7 [135.2–139.7]	137.2 [133.0–139.8]	139.6 [135.5–143.8]	0.090
Serum potassium, mmol/L	4.0 [3.4–4.3]	3.1 [2.7–3.9]	3.1 [1.9–4.1]	0.051
Serum phosphate, mmol/L	1.6 [1.3–1.7]	1.1 [0.8–1.3]	1.2 [0.7–2.3]	0.020
Serum chloride, mmol/L	103.2 [100.7–106.6]	102.6 [97.5–115.5]	112.2 [111.3–113.7]	0.053
Serum magnesium, mmol/L	0.9 [0.7–1.0]	0.9 [0.8–1.0]	0.9 [0.8–1.1]	0.476
Alanine aminotransferase, U/L	22.9 [15.9–29.8]	31.8 [23.5–90.8]	12.6 [8.3–58.6]	0.042
Lactate dehydrogenase, U/L	245.6 [184.3–282.3]	200.6 [179.3–263.0]	343.4 [250.9–675.2]	0.045
Serum creatinine, μmol/L	31.0 [21.5–39.7]	40.2 [29.5–48.8]	40.5 [29.9–83.8]	0.134
Blood urea nitrogen, mmol/L	4.2 [3.3–5.3]	5.0 [3.2–9.3]	4.8 [3.0–9.4]	0.438
pH	7.4 [7.3–7.5]	7.4 [7.3–7.5]	7.3 [7.2–7.4]	0.441
Hemoglobin, g/L	118.0 [106.0–129.5]	91.0 [79.5–107.3]	112.0 [99.5–128.0]	0.003
Persistent NC, *n* (%)	8/11 (72.7)	4/5 (80)	6/10 (60)	0.747

NC, nephrocalcinosis; CSC, corrected serum calcium.

### Etiological factors

As demonstrated in Table 3, the underlying etiologies of NC varied among the patients. RTA was the most frequent cause, accounting for 24% of the cases. Other notable etiologies included malignancies (8%), IgA Vasculitis with nephritis (6%), and hypophosphatasia (4%). Additionally, 26% of the cases had unknown etiologies, with 3 patients diagnosed with genetic syndromes where the exact cause remained unidentified. However, it was interesting to note that, all the 3 cases presented with growth and development retardation as the core manifestation. Two of them were siblings, both presenting with special facial features and hypercalcium crises, and they were eventually suspected of having familial hypocalciuric hypercalcemia (FHH). The other child was accompanied by skin pigmentation and hirsutism (Table 3).

**Table 3:** Etiologies related to NC.

Etiology	*N* (%)
Renal tubular acidosis	12 (24)
Malignancies	4 (8)
IgA vasculitis with nephritis	3 (6)
Hypophosphatasia	2 (4)
Urinary tract infections	2 (4)
Autosomal dominant hypocalcemia type I	1 (2)
Hypothyroidism	1 (2)
Glycogen storage disease	1 (2)
Williams syndrome	1 (2)
Congenital adrenal hyperplasia	1 (2)
Osteogenesis imperfecta	1 (2)
Mucopolysaccharidosis type IV	1 (2)
Idiopathic hypercalciuria	1 (2)
Dent's disease type I	1 (2)
Bartter syndrome	1 (2)
Medullary sponge kidney	1 (2)
Donohue's Syndrome	1 (2)
Hereditary spherocytosis	1 (2)
Nephrotic syndrome	1 (2)
Unknown etiology	13 (26)

NC, nephrocalcinosis; PTH, parathyroid hormone.

### Genetic analysis

**Figure 1:**
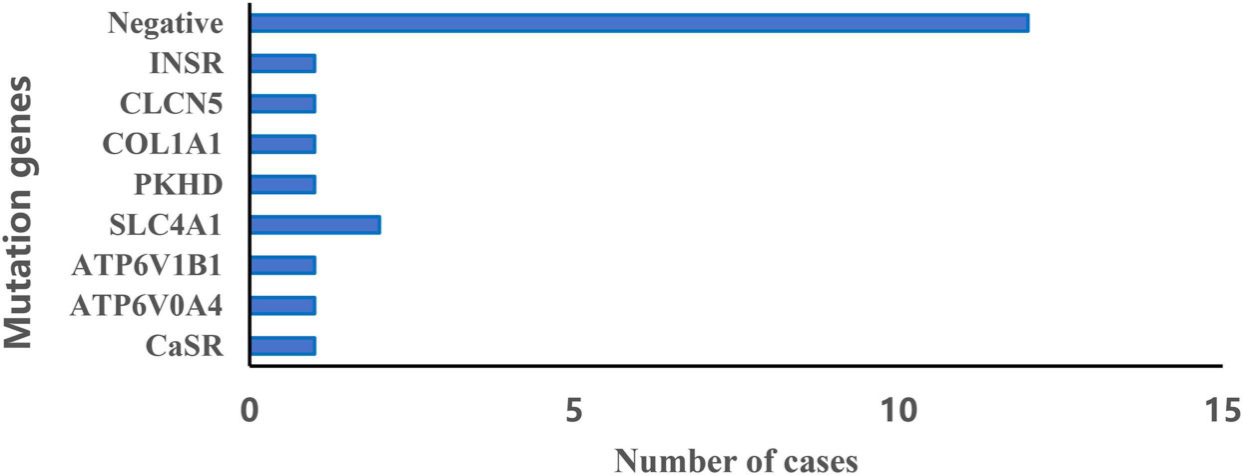
Distribution of genetic diagnoses with nephrocalcinosis.

The genetic analysis revealed a variety of mutations associated with NC (Figure 1). Most notably, a significant portion of the cases (12 out of 22, 55%) did not have any detectable mutations. Among those with identified mutations, the most commonly affected gene was SLC4A1 in two patients. Since the loss of the genetic report for one case diagnosed with Williams syndrome via whole exome sequencing (WES), we were unable to list the specific genetic result in Figure 1. This indicates a diverse genetic landscape in NC, with multiple genes potentially contributing to the pathogenesis.

### Follow-up results

The median follow-up time was 47.5 (range 3–105) months. All patients diagnosed with NC received treatment with intravenous fluids and targeted etiological therapies. Follow-up abdominal ultrasonography was available on 26 patients, revealing that 18 (69%) showed no change in their NC status, indicating persistent NC. 2 of them later developed NL, with 1 patient requiring surgical intervention. Among the patients with improvement, one child with leukemia responded to potassium citrate therapy, whereas the remaining five improved with etiological treatment alone. In contrast, among the 20 patients without improvement, 60% had an underlying genetic disorder, suggesting a poorer radiologic prognosis in genetically mediated nephrocalcinosis.

Renal function was monitored in 23 patients during follow-up. Of these, 20 (87%) maintained normal renal function, while 3 patients (13%) developed renal impairment. 2 patients had only mild elevations in blood urea nitrogen, and 1 patient with mucopolysaccharidosis progressed gradually to stage 3 CKD.

## Discussion

In this retrospective study, we provide comprehensive insights into the clinical and genetic landscape of NC in children, underscoring the predominance of genetic and metabolic disorders in its etiology. A significant proportion of cases were detected incidentally, reflecting the often subtle or nonspecific clinical presentation of NC in the pediatric population.

Our findings align with previous studies indicating that NC is more prevalent in younger children, particularly within the first year of life, and that males appear to be more frequently affected (18, 19). In this study, half of the cases were diagnosed before the age of two, with infants constituting the majority. The male predominance in this study (62%) further confirmed that male children might be more susceptible to NC. The incidental discovery of NC in up to 72% of cases underscores the potential for underestimation of its true prevalence and incidence, pointing to the importance of early ultrasonographic screening in pediatric populations, which may facilitate timely intervention before irreversible renal damage occurs (3).

The clinical features of NC tend to be diverse and nonspecific, ranging from nausea and vomiting to more severe symptoms such as polyuria and developmental delays (20). Nevertheless, our study and the previous reports showed that the most prominent clinical manifestation was failure to thrive (20, 21), followed by nausea-vomiting, abdominal pain and polyuria in our study. This finding highlights the critical need for early genetic evaluation in children with NC and associated developmental issues, as early diagnosis and intervention are crucial in preventing long-term disabilities. For families in whom a causative genetic mutation has been identified, genetic counseling should include discussing the inheritance pattern, providing the opportunity to screen other family members, and offering pre-implantation genetic diagnosis (PGD) testing for subsequent pregnancies (22).

The kidneys play a vital role in maintaining calcium homeostasis by regulating the reabsorption and excretion of calcium in response to changes in blood calcium levels and hormonal signals (23). Disruptions these mechanisms can lead to disorders such as hypercalcemia or hypocalcemia, which can significantly affect various organ systems and overall health (24). In our study, children with NC were categorized into three subgroups based on their total serum calcium levels. Unfortunately, the radiological improvement rate of NC remained unsatisfactory across all groups, suggesting that calcium imbalance alone may not fully account for disease persistence. Notably, among the hypercalcemic group, 75% (9/12) presented symptoms within the first year of life and frequently exhibited with mild to moderate anemia. However, the relationship between serum calcium levels and the progression of NC remains to be further elucidated.

Among all etiologies of calcium deposition in adults, environmental triggers are the main causes, while our findings support the view that childhood NC was mainly influenced by genetic and metabolic disorders (54%) (9). Among patients with identifiable etiologies, 73% (27/37) of them suffered from genetic and metabolic disorders, and 20% had a positive family history of nephrolithiasis, RTA and kidney cyst. Consistent with previous studies, renal tubular acidosis is the most common cause among all genetic metabolic diseases (21). RTA is a tubulopathy that affects multiple organ systems either because of defects in genes that share expression between kidney and other organs or because acidosis affects extrarenal systems (25). Patients with RTA may be identified by family screening before overt symptoms become apparent or present later in childhood with growth failure or in adulthood with urolithiasis (26). Among children diagnosed with RTA, genetic mutations were identified in four cases. The two patients with *SLC4A1* mutations exhibited a later onset age (8 months and 7 years, respectively) compared with those carrying *ATP6V1B1* and *ATP6V0A4* mutations (1 month and 3 months, respectively) (27). In this study, we found the median age at the time of diagnosis of RTA was 37.4 (range 1–99) months, which was also consistent with the previous study (27, 28).

The lack of comprehensive genetic data for all patients, primarily due to economic factors and variations in parental awareness, represents a significant limitation in fully understanding the genetic underpinnings of NC. Meanwhile, some of them got genetic testing at external medical institutions resulting in the unavailability of specific genetic information. Nevertheless, our findings reflect a higher prevalence of monogenic causes in children compared with adults (20.8% vs. 11.4%) (29). This is concordant with the findings from Joung et al., who reported that the monogenic mutation rates were much higher in full-term children (1).

Among children who had genetic testing in our study, 45% of them exhibited genetic abnormalities, and mutations in these genes may be closely related to the disease. For example, RTA had been reported to be related to the *SLC4A1*, *ATP6V1B1* and *ATP6V0A4* genes, and mutations in *ATP6V1B1* and *ATP6V0A4* genes were predisposed to causing sensorineural hearing loss in children (30). It is widely recognized that Dent's disease is associated with a mutation in the *CLCN5* gene, leading to proximal tubular dysfunction (31). Researches have shown that mutations in the *PKD* and *CaSR* genes can cause a variety of kidney-related diseases (32, 33). In an infant, Vankevičienė K et al. confirmed osteogenesis imperfecta using *COL1A1* gene variant detection via exome sequencing (34). *INSR* gene dysfunction is associated with nephrocalcinosis (35). A 6-year-old Paraguayan girl was diagnosed with Rabson-Mendenhall syndrome (RMS), who presented with hypertrichosis, acanthosis nigricans and nephrocalcinosis. Genetic testing by next-generation sequencing (NGS) revealed two pathogenic variants in exons 2 and 19 of the *INSR* gene (36). Notably, all the these genetic mutations were identified in our study, supporting the notion that NC frequently represents a renal manifestation of systemic genetic syndromes. The varied diagnoses and treatments resulting from these distinct genetic mutations underscore the significance of genetic testing in managing and predicting the prognosis of pediatric NC.

The persistence of NC for a long time raised concerns about irreversible kidney damage in these individuals (37). Most published reports focused primarily on the prompt management and clinical features, with few studies addressing the prognosis of NC. In a Korean study that included 464 children with NC (Preterm: Full-term = 349: 115), 62% of patients experienced resolution of NC (1). However, in our study, only a small proportion of patients (23%) showed improvement. Prematurity and use of furosemide, vitamin D, and steroids were clinical risk factors of NC (10, 38, 39). The primary etiologies of NC in preterm infants are iatrogenic and transient, particularly related to the use of furosemide and immature renal function, and most of them have good prognoses (40, 41). Hence, we hypothesize that the poor improvement rate in this study may be associated with the small proportion of preterm infants.

Although most cases in our study maintained normal renal function during follow-up, 13% developed renal impairment, and one progressed to stage 3 CKD. These findings are consistent with reports that NC may persist for years and, in some cases, lead to progressive renal dysfunction. Similarly, a previous 5-year study containing 44 children with hypervitaminosis D-related NC demonstrated persistent calcifications with no radiologic resolution (37). However, given that only a minority progressed to CKD, the precise relationship between persistent nephrocalcinosis and long-term renal outcomes remains uncertain and warrants further studies.

The present study has several limitations due to its retrospective nature. Referral bias likely exists as patients were enrolled from hospitalized children in a tertiary academic center. Additionally, selection bias is possible, as clinicians did not perform genetic testing on all patients with NC, only on those with suspected genetic causes. Furthermore, the study did not include a classification of NC based on the location and extent of calcium deposition observed via ultrasonography, which could have provided more precise insights into disease severity and prognosis. Lastly, the study faced follow-up bias, with some patients not completing regular abdominal imaging and renal function tests, limiting the accurate assessment of long-term renal function.

## Conclusions

NC in children is a potentially overlooked condition that can lead to kidney stones or chronic renal insufficiency. However, the relationship between NC and severe complications, such as CKD and ESRD, requires further investigation. Congenital or genetic diseases may be the main systemic diseases leading to NC. Genetic testing can provide important clues for the diagnosis and prognosis of these conditions and may significantly help in managing persistent NC.

## Data Availability

The original contributions presented in the study are included in the article/Supplementary Material, further inquiries can be directed to the corresponding author.
